# Psychological Profile and Social Behaviors of Patients with Hyperacusis

**DOI:** 10.3390/jcm11247317

**Published:** 2022-12-09

**Authors:** Luca Sacchetto, Enrico Apa, Andrea Ciorba, Silvia Palma, Valeria Caragli, Chiara Gherpelli, Daniele Monzani, Elisabetta Genovese, Riccardo Nocini

**Affiliations:** 1 Ear Nose and Throat (ENT), Department of Surgical Sciences, Dentistry, Gynecology and Pediatrics, University of Verona, 37134 Verona, Italy; 2 Audiology, ENT Department, University of Modena and Reggio Emilia, 41100 Modena, Italy; 3 ENT & Audiology Unit, Department of Neurosciences, University Hospital of Ferrara, Via Aldo Moro 8, 44124 Cona, Italy; 4 Audiology, Primary Care Department, Ausl Modena, 41121 Modena, Italy

**Keywords:** hearing loss, hyperacusis, somatization, anxiety, social phobia

## Abstract

Increased noise sensitivity refers to the abnormal subjective response to external sounds, with a prevalence of between 8% and 15.2% in the adult population as suggested by epidemiological studies. The basic neural mechanism of hyperacusis still remains obscure, so therapies for this often-devastating symptom remain elusive. The aim of this study was to assess psychological profiles in patients with presbycusis without tinnitus in a perspective case-control design. All subjects were initially submitted to audiological evaluation (tympanometry, recordings of the acoustic reflex thresholds, pure tone audiometry) and subsequently were administered the following questionnaires: the hyperacusis questionnaire (HQ), the brief symptom inventory (BSI), and the modified somatic perception questionnaire (MSPQ). Patients with hyperacusis reported a total score and subscales (attentional, social, and emotional) of the HQ significantly higher than controls. They also reported higher scores of the MSPQ and significantly higher mean values with concern to the somatization, obsessive-compulsive, interpersonal sensitivity, depression, and anxiety subscales of the BSI. These results show that psychological distress, as expressed by higher level of somatic attention, somatization, anxiety, and depression, is a significant factor to consider for a complete diagnosis and effective treatment of hyperacusis. For a correct diagnosis of patients seeking help for hyperacusis, their psychological distress should also be assessed, regardless of their hearing abilities. Further studies are required to investigate the pathological mechanisms that are involved in the onset of hyperacusis in patients with normal hearing and those with sensorineural hearing loss.

## 1. Introduction

Increased noise sensitivity refers to the abnormal subjective response to external sounds. It is considered to be an individual trait that does not necessarily depend on hearing loss [1] even if noise exposure and consequent noise-induced hearing loss are considered major risks in many cases [2]. It could be considered an abnormal loudness perception relative to environmental sounds that are ordinarily well-tolerated or even ignored by most individuals [3]. Differently from phonophobia, which typically refers to sensitivity to loud sounds such as that associated to migraine, and misophonia [4], which indicates an abnormal emotional reaction (fear, aversion, autonomic “fight or flight” reaction) to specific sounds (for example, fingernails scratching against a chalkboard or a person’s chewing), hyperacusis refers to an individual’s reduced tolerance to sounds in general. It should also be distinguished from loudness recruitment which indicates a faster increase of loudness perception with respect to a less rapidly rising intensity of external sounds.

The prevalence of hyperacusis in adults is not yet definitively stated, but two epidemiological studies suggest its rates lie between 8% and 15.2% in the adult population; it is significantly higher in women and in people with hearing disorders over those with normal hearing [5,6,7]. Various occupations (musicians, music students, teachers, and others) have been found to be high risk factors for hyperacusis [8]. Four subtypes of hyperacusis were proposed according to its main clinical feature: excessive loudness, annoyance, fear, and pain [2] where sounds are perceived uncomfortably loud, unpleasant, frightening, or painful, respectively. While in clinical practice it may not be easy to disambiguate these categories, this classification could be an advantage for neurophysiological research [9]. The basic neural mechanism of hyperacusis still remains obscure, so that therapies for this often-devastating symptom remain elusive [10]. A reduced tolerance to sounds is observed not only in patients with hearing loss, but also across different neurophysiological disorders, such as William’s syndrome, Lyme disease, Addison’s disease, head injury, migraine, and multiple sclerosis [2]. Decreased sound tolerance is also the most prevalent, persistent, and disabling sensory features of autism [11].

These observations suggest excessive gain enhancement in response to peripheral auditory stimuli in accordance with the so-called “Central Gain Model” [12]. According to this model, some human imaging data indicate that hyperacusis is associated with enhanced sound-evoked activity in multiple-auditory processing centers, namely auditory cortex, medial geniculate body, and inferior colliculus, despite the absence of clinically evident hearing loss [13]. On the other hand, hyperacusis has been reported in patients with post-traumatic stress disorders [14], anxiety disorders, and anxiety-related personality traits [15] and depression [6,16]. Avoidance behavior toward self-exposure to everyday sounds was also documented and contributes to worsen psychological well-being of patients with hyperacusis [17]. It should be noted that the aforementioned studies included many patients who also complained of tinnitus, a well-known source of anxiety and depression per se [18], so that the link between psychological distress and hyperacusis alone can be hardly isolated. To our knowledge, a more detailed description of psychological distress in patients with hyperacusis alone is confined to a single case report [19]. Therefore, the aim of this study was to assess psychological profile in patients with presbycusis without tinnitus in a prospective case-control design.

## 2. Material and Methods

This perspective study was based on data collected from patients recruited at the University Hospitals of Modena and Ferrara between September 2021 and September 2022. The subjects, all adults, suffered from hyperacusis (ICD-10, H93.2–Abnormal auditory perceptions).

Exclusion criteria were: the presence of tinnitus, middle ear dysfunction, and neurological or psychiatric diseases that could prevent them from answering the self-administered questionnaires and easily participating in the medical examination.

Patients undergoing any pharmacological treatment and/or using assistive devices for hearing loss at the time of examination were also excluded.

Controls were recruited from medical and staff personnel and relatives accompanying patients to the visits. The study was approved by the Institutional Ethic Committee (n°228/2021) and conformed to the standards set by the Declaration of Helsinki. Eligible participants and controls gave their written consent before enrollment in the study.

### 2.1. Audiological Evaluation

All subjects were initially submitted to otoscopy, tympanometry, and recordings of the acoustic reflex thresholds (ART). Pure tone audiometry air conduction thresholds at frequencies 250, 500, 1000, 2000, 4000, and 8000 Hz were obtained for each ear separately, in a sound-treated booth so to provide a standardized measure of the weakest sounds detectable, for tones in the range of human audible frequencies. Air and bone conduction were tested in all cases. Average four-frequency pure-tone audiometry (PTA) was computed by using the average from the thresholds in the better ear. Hearing loss was defined as a PTA of 20 dB or higher in the better ear, in accordance with a recent recommendation [20]. Thereafter, a modified pure-tone audiometry was performed to measure loudness discomfort levels (LDLs). LDLs predict the level at which tones are perceived by a patient to be uncomfortably loud. LDLs’ estimate of loudness discomfort is proved to be an efficient and valid clinical measure for characterizing the “threshold of discomfort” [21]. In order to obtain LDLs, continuous pure-tone signals were presented for three seconds, with a two-second interval between each presentation. The initial stimulus intensity was at 50 dB and was presented by ascending steps of 5 dB each, until the subject complained of their initial discomfort with the loudness. The average LDL of normal-hearing individuals is approximately 100 dB HL and LDLs of less than 80–85 dB HL can be considered as abnormally low [21].

### 2.2. Psychometric Questionnaires

After the audiological section, all subjects (patients and controls) were administered the following questionnaires:

The ***hyperacusis questionnaire (HQ)*** was developed by Khalfa et al. [22] to address adaptive, cognitive, and emotional reactions to sound perceived as uncomfortably loud. The questionnaire is divided into two parts. The first includes three binary questions about the presence of auditory disorders, the past or present exposure to noise in general, and the possibility about a decreased tolerance to noise over time. The second part consists of 14 items that will be scored over three major dimensions: attentional (questions 1–4), social (questions 5–10), and emotional (questions 11–14). Answers to each question/ item are given on a 4-point scale, ranging from ‘no’ (scoring 0 points), ‘yes, a little’ (scoring 1 point), ‘yes, a lot’ (scoring 2 points) to ‘yes, quite a lot’ (scoring 3 points). Total score ranges from 0 indicating no hyperacusis to 42 spelling the worst level of the symptom. An Italian validation study showed good internal consistency of the scale as documented by Cronbach’s α coefficient = 0.89 [23].

The ***brief symptom inventory (BSI)*** contains 53 items [24]. The instrument provides information on overall psychological distress, across 9 symptom domains. These include somatization, obsessive-compulsivity, interpersonal sensitivity, depression, anxiety, hostility, phobic anxiety, paranoid ideation, and psychoticism. Three composite scores can be computed and include the general severity index (GSI), the positive symptom total (PST), and the positive symptom distress index (PSDI).

In the framework of this study, only GSI is reported because it combines information about the number of symptoms and the intensity of distress and, therefore, is considered the most sensitive indicator of the patient’s overall distress level. Patients are asked to rate each of the 53 items on a five-point Likert scale (0–4), ranging from not at all (0) to extreme (4). Dimension scores are calculated by summing the values for the items included in that dimension and dividing by the number of items endorsed in it. GSI is calculated using the sums for the nine dimensions plus the four additional items not included in any of the dimension scores, and dividing by the total number of items to which the individual responded. When answering, subjects are asked to consider the relevance of each item to their experience in the past 7 days, including the day of examination.

The ***modified somatic perception questionnaire (MSPQ)*** [25] is a 13-item self-report scale for patients with chronic pain or disabilities. It can help identify somatic complaints that may be associated with psychological responses such as anxiety or depression. It explores the occurrence in the last week of various vegetative symptoms such as heart rate increase, nausea, dizziness, sweating, or feeling faint. Each item is answered using a Likert scale with 4 possible responses ranging from “not at all” (0 points) to “could not have been worse” (3 points). The total score (sum score of all 13 items) ranges from 0 to 39. The higher the score the more marked the general somatic symptoms.

All questionnaires were filled in by patients in a self-administered way.

### 2.3. Statistical Analysis

Variables were described as frequencies or mean values and standard deviations. In the comparison between patients and controls, χ2 and *t*-test were used to analyze categorical and non-categorical variables respectively, with significance level of *p* < 0.05. All statistical analyses were performed using SPSS v. 26.0 (SPSS Inc., Chicago, IL, USA).

## 3. Results

The age of the hyperacusis patients (*n* = 35) ranged from 23 to 59 years (mean age = 44, SD = 11.11), 15 of them were men (42.9%). Controls (*n* =37) were between 20 and 61 years old (mean age = 47.6, SD = 10.7) and 17 of them were men (45.9 %). The two groups did not differ statistically according to age (t 0.51; df = 220; *p =* 0.61) or gender (ϰ^2^ = 0.069; df = 1; *p* = 0.18). Educational level, expressed by years of study, did not significantly differ between patients (mean = 13.4 years, SD = 3.9) and controls (mean = 11.8 years, SD = 3.8) (t = −1.76, df = 70, *p* = 0.82).

Eighteen controls and nineteen patients reported in the HQ test a general exposure to noise, i.e., it was about the same percentage in both groups (ϰ^2^ = 6.27, df = 1, *p* = 0.72). The increase of a reduced tolerance to sounds over time was reported by only three controls and thirty-two patients (ϰ2 = 49.9, df = 1, *p* < 0.0001).

Five (14.3%) patients with hyperacusis and ten (27%) controls were also affected by hearing loss.

Hearing loss, on average, was mild and limited to high frequencies in all cases (hearing threshold between 20 and 40 dB at 4000–8000 Hz). The results of the examinations are displayed in Table 1.

**Table 1:** Mean scores and standard deviations of pure tone audiometry and PTA (expressed in decibel) are displayed for both ears. The significance level is reached for *p* values < 0.05 and <0.005.

	Total Sample		Hyperacusis Cases		Controls		Independent *t*-Test		
	Mean	S.D.	Mean	S.D.	Mean	S.D.	t	df	*p*
Hearing Threshold (dB)									
500 Hz right	14.03	6.79	14.43	7.84	13.65	5.73	−0.48	70	0.63
1000 Hz right	13.06	6.53	12.29	6.49	13.78	6.49	0.97	69	0.33
2000 Hz right	15.76	8.62	14	7.35	17.43	9.74	1.71	70	0.09
4000 Hz right	22.99	11.65	10.57	10.11	25.27	12.01	1.72	70	0.09
8000 Hz right	29.65	19.63	25.29	17.69	33.78	20.69	1.87	70	0.07
500 Hz right	19.79	17.06	22.94	20.60	16.89	12.6	−1.47	69	0.14
1000 Hz left	17.51	17.07	20.59	18.82	16.88	15.33	−0.91	69	0.37
2000 Hz left	22.96	19.35	24.71	23.96	21.35	14.02	−0.72	69	0.47
4000 Hz left	31.83	21.81	32.65	25.82	31.08	17.68	−0.29	69	0.76
8000 Hz left	36.30	25.18	31.09	40.81	40.81	25.83	1.62	67	0.11
PTA right	16.46	6.45	15.32	6.13	17.53	6.64	1.46	70	0.15
PTA left	23.31	17.66	25.22	21.53	21.55	13.21	−0.87	69	0.39

LDLs in patients with hyperacusis were all significantly lower in both ears with respect to controls. LDLs for each hearing frequency in the two groups are displayed in Table 2.

**Table 2:** Mean scores and standard deviation of LDLs (loudness discomfort levels) expressed in decibel are displayed for both ears. The significance level is reached for *p* values < 0.05 and <0.005 (**).

	Hyperacusis Cases		Controls		Independent *t*-Test		
Frequencies (Hertz)	Mean	S.D.	Mean	S.D.	t	df	*p*
**Right ear**							
500 Hz	82.3	4.7	94.2	10.2	6.2	71	<0.0001 **
1000 Hz	84.1	3.3	93.5	9.9	5.6	72	<0.0001 **
2000 Hz	85.1	3.2	92.7	7.7	5.7	70	<0.0001 **
4000 Hz	84.7	4.5	93.6	6.6	6.91	71	<0.0001 **
**Left ear**							
500 Hz	84.1	4.8	94.4	9.6	6.31	72	<0.0001 **
1000 Hz	84.2	5.5	96.5	7.6	6.94	72	<0.0001 **
2000 Hz	83.7	5	96.3	7	6.83	71	<0.0001 **
4000 Hz	84.1	4.9	97.8	8.4	7.72	72	<0.0001 **

On the contrary, the ARTs were not different between patients and controls in both ears. ART for each hearing frequency (range: 500–4000 Hz) in the two groups are displayed in Table 3.

**Table 3:** Mean scores and standard deviation of ART expressed in decibel, for each frequencies tested are displayed for both ears. The significance level is reached for *p* values < 0.05 and <0.005.

	Hyperacusis cases		Controls		Independent *t*-Test		
Frequencies (Hertz)	Mean	SD	Mean	SD	t	df	*p*
**Right ear**							
500 Hz	86.6	±6.1	86.6	±6.0	−0.62	71	0.536
1000Hz	89.4	±5.7	88.9	±6.7	−0.31	70	0.756
2000 Hz	89.1	±5.3	87.4	±7.8	−1.13	72	0.263
4000 Hz	92.3	±8.1	90.3	±7.6	−1.17	71	0.244
**Left ear**							
500 Hz	87.3	±5.6	87.9	±5.5	−0.73	70	0.469
1000 Hz	91.0	±6.6	87.9	±5.8	−1.78	71	0.081
2000 Hz	90.8	±5.8	88.2	±7.7	−1.39	72	0.168
4000 Hz	92.6	±8.4	89.2	±8.2	−1.58	72	0.118

Patients with hyperacusis reported a total score and subscales (attentional, social, and emotional) of the HQ significantly higher than controls (*p* < 0.05). They also reported higher scores of the MSPQ than controls (*p* < 0.0001). Furthermore, patients reported significantly higher mean values than controls with concern to somatization (*p* < 0.05), obsessive-compulsiveness (*p* < 0.001), interpersonal sensitivity (*p* < 0.05), depression (*p* < 0.005), and anxiety (*p* < 0.05) subscales of the BSI, with a GSI considerably higher in patients than in controls (*p* < 0.005). The results of the psychometric assessment (HQ, MSPQ, and BSI) of hyperacusis patients and controls are displayed in Table 4.

## 4. Discussion

This study compared the results of 35 patients with a primary complaint of hyperacusis without tinnitus and their audiological data to those of 37 controls, well matched for sex, age, and educational level. In agreement with previous studies, the LDLs were significantly lower (about 85 dB), in both ears, for the hyperacusis patients, when compared to those of the control group [26,27]. The LDLs level in control subjects were around 95 dB, which is a little lower than those reported in a normative study [20]. We hypothesized that the discrepancy could be due to the different mean age of controls we examined, which was much higher than the mean age of subjects enrolled in the previous study [27]. As already reported, the LDL hardly varies with frequency in the range from 500 to 4000 Hz in both ears [27]. On the contrary, patients with hyperacusis recruited in this study showed LDLs closely resembling those from a normative study by Sherlock and Forby [21]. The ARTs, on average, were about 90 dB in both ears and no difference was found between both groups as well as concerning the PTA. Taken together, these results suggest that hyperacusis could be detected only by determining the LDLs and that it is more probably due to a central rather than a peripheral dysfunction of the auditory system [28].

Interestingly, patients exhibited high levels of somatic attention and were more hypervigilant to bodily sensations than controls, as suggested by the MSPQ. This increased somatic awareness is probably the reason for their attention disruption, revealed by the attentional subscale of the HQ [29]. Similarly to somatoform disorders, where a person experiences bodily symptoms that cannot be accounted for by a medical or neurological diagnosis, patients with hyperacusis exhibit a more pronounced tendency to somatization than controls, as evidenced by the BSI. In other words, they are particularly prone to experience sensations as intense, noxious, and disturbing [30]. Together with a tendency to somatization, they also showed higher levels of anxiety and depression than controls, in analogy to patients with somatoform disorders who frequently are affected by comorbid anxiety or depressive disorders [31]

Subjects with hyperacusis exhibited higher scores of the interpersonal sensitivity subscale of the BSI than controls. Interpersonal sensitivity refers to the accuracy and/or appropriateness of perceptions, judgments, and responses individuals have with respect to one another, including criticisms and rejections [32]. Individuals with high interpersonal sensitivity tend to be hypersensitive to negative evaluation from others, which causes them social avoidance and distress [33,34]. This may contribute to social avoidance behavior in relation to noisy recreational activities as documented by higher scores of the HQ social subscale reported by patients with hyperacusis with respect to controls. Patients also reported higher scores of the obsessive-compulsive and anxiety subscales of the BSI than controls as already shown by Schwartz [35] and Aazh [36], although a causal relationship between them and hyperacusis is still to be established. Subjects with hyperacusis reported higher levels of depression than controls and this observation can be explained by previous results that suggested both depression and hyperacusis can be caused by 5-hydroxytryptamine hypoactivity in the brain [37]; the enhancement of 5-HT activity due to imipramine [38] and lithium prescribed for depression also play a role in alleviating hyperacusis symptoms [39].

Finally, psychological distress is more pronounced in patients with hyperacusis than in controls, as suggested by the higher score of the global severity index in the former.

The causality of this association is not well-established. One experiment showed that women with high emotional exhaustion levels developed hyperacusis after an acute stress task [40]. This result may suggest that abnormal auditory sensations are consequences of a psychosomatic effect. An increased LDL test will not detect signs of hyperacusis in women with high levels of emotional exhaustion if they are not acutely stressed during the testing, so that these results could not be extended to chronic conditions. On the other hand, intolerance to sound could be a stressful condition due to avoidance behaviors and social isolation so that stress, anxiety, and depression are consequences. Therefore, it is plausible that the association is bi-directional, i.e., that hyperacusis is stressful and that stress causes increased sensitivity to sounds in a real-life acoustic scenario.

This study has some limitations. We only tested pure tone stimuli to determine to which extent patients and controls reported unbearable levels of loudness. For the specific group of patients we focused on, it would be good to measure (in a follow-up study) otoacoustic suppression emissions.

Overall, it is shown that psychological distress, as expressed by higher levels of somatic attention, somatization, anxiety, and depression, is a significant factor to consider for a complete diagnosis and effective treatment of hyperacusis. Thus, patients seeking help for hyperacusis, regardless of normal hearing function, should also be assessed for psychological distress for a correct diagnosis. Further studies are needed to address which pathological mechanisms are involved in the onset of hyperacusis, both in normal subjects and in patients affected by sensorineural hearing loss.

## Figures and Tables

**Table 4:** Mean scores and standard deviation of HQ, MSPQ, and BSI. The significance level is reached for *p* values < 0.05 (*) and <0.005 (**).

	Hyperacusis Cases		Controls		Independent *t*-Test		
	Mean	S.D.	Mean	S.D.	t	df	*p*
HQ total score	24.2	7.2	5.51	4.3	−13.5	72	<0.0001 **
Attentional subscale	6.69	2.3	1.95	1.8	−9.8	72	<0.0001 **
Social subscale	9.49	3.4	1.76	1.7	−12.4	72	<0.0001 **
Emotional subscale	7.97	2.4	1.81	1.7	−12.8	72	<0.0001 **
MSPQ	15.03	10.5	6.35	6.4	−4.2	72	<0.0001 **
Somatization	0.97	0.8	0.44	0.7	−2.9	72	0.005 *
Obsession-compulsion	1.20	1.0	0.38	0.6	−4.0	72	<0.0001 **
Interpersonal sensitivity	0.74	1.1	0.24	0.6	2.4	72	0.018 *
Depression	0.86	0.9	0.3	0.6	−3.0	72	0.003 **
Anxiety	1.06	1	0.38	0.8	−3.1	72	0.002 **
Hostility	0.54	0.6	0.3	0.6	−1.8	72	0.082
Phobic anxiety	0.45	0.8	0.16	0.5	−1.8	72	0.076
Paranoid ideation	0.74	0.9	0.38	0.7	−2.0	72	0.052
Psychoticism	0.49	0.9	0.16	0.5	−1.9	72	0.059
Global Severity Index	0.83	0.7	0.22	0.5	−4.1	72	>0.0001 **

## Data Availability

The data presented in this study are available from the corresponding author on request due to privacy restrictions.
